# Pioglitazone and thyroid cancer risk in patients with type 2 diabetes

**DOI:** 10.1186/2049-3002-2-S1-P77

**Published:** 2014-05-28

**Authors:** Chin-Hsiao Tseng

**Affiliations:** 1 Department of Internal Medicine, National Taiwan University College of Medicine, Taipei, Taiwan

## Background

The association between pioglitazone use in patients with type 2 diabetes and thyroid cancer risk has not been investigated.

## Materials and methods

The reimbursement data of all diabetic patients treated with oral anti-diabetic agents or insulin within the period from 1996 to 2009 were retrieved from the Taiwan’s National Health Insurance. An entry date was set at 1 January 2006 and the incidence of thyroid cancer was followed up until the end of 2009 in 1097215 patients with type 2 diabetes. Incidences for ever-users, never-users and subgroups of dose-response exposure to pioglitazone (i.e. tertile cutoffs of cumulative duration and cumulative dose) were calculated. Cox regression was used to estimate the hazard ratios after adjustment for age, sex, diabetes duration, comorbidities (hypertension, obstructive pulmonary disease, stroke, ischemic heart disease, peripheral arterial disease, eye disease, obesity, dyslipidemia, previous thyroid benign disease, and other cancer), medications (statin, fibrate, angiotensin-converting enzyme inhibitor and/or angiotensin receptor blocker, calcium channel blocker, sulfonylurea, metformin, insulin acarbose, rosiglitazone, aspirin, ticlopidine, clopidogrel, dipyridamole and non-steroidal anti-inflammatory drugs) and potential detection examinations (thyroid sonography, thyroid aspiration, and/or thyroid function test).

## Results

There were 58378 ever-users and 1038837 never-users, with respective number of incident thyroid cancer: 48 (0.08%) and 921 (0.09%). Neither the overall hazard ratio nor any of the dose-response parameters showed significant association.

## Conclusions

Pioglitazone use has a null association with thyroid cancer risk.

